# Spatial-Temporal Accessibility and Inequality of Veterinary Service in Hong Kong: A Geographic Information System-Based Study

**DOI:** 10.3389/fvets.2022.857914

**Published:** 2022-04-15

**Authors:** Ka Yiu Ng, Chun Long Ho, Keumseok Koh

**Affiliations:** Department of Geography, The University of Hong Kong, Hong Kong, Hong Kong SAR, China

**Keywords:** veterinary care, spatial accessibility, Geographic Information System (GIS), Hong Kong, affordable care, companion animals, animal welfare

## Abstract

Veterinary services are vital to the welfare of pets and their owners. Previous studies examined multiple factors affecting pet owners' decision to consult veterinarians, yet few studied the spatial accessibility of veterinary services. This study is one of the pioneering studies on the spatial-temporal accessibility of veterinary service and how it is associated with social and spatial inequality in Hong Kong. We measured the spatial availability and accessibility of both general and 24/7 veterinary clinics (i.e., veterinary clinics offering service for 24 hours, seven days a week or providing emergency services outside of business hours) using Geographic Information System and principal component analysis. We found that the spatial distribution pattern of general and 24/7 veterinary clinics can be explained by the average district-to-district distances and the area of a district. In addition, social and spatial inequality of access to veterinary services were observed. The spatial accessibility of general veterinary clinics within walking distance is negatively correlated with household size and the number of public-housing and subsidized-housing households, but positively correlated with the number of private-housing households. The spatial availability and accessibility of 24/7 veterinary service are positively correlated with the number of private housing households and households with the highest monthly household income, and the latter also positively correlates with a population with a post-secondary degree, further shedding light on the social and spatial inequality issue that communities with wealthier households and highly educated populations have more accessibility to 24/7 veterinary services. Last, we argue that the need-based veterinary support tends to target remote rural areas while overlooking the new growth areas close to the traditional urban core but poor in accessibility to veterinary care. Therefore, a comprehensive investigation into the pet ownership landscape and their needs over space and time will be beneficial to construct a more robust animal welfare system in Hong Kong.

## Introduction

Companion animals or pets have long been in a close relationship with human beings and the positive effect of their companionship on humans' well-being is well documented (1, 2). The presence of pets is beneficial to peoples' mental well-being. For instance, the influence of good human-pet compatibility was found to be positive on one's mental health by relieving anxiety and distress (3). Other than mental well-being, the positive effect of pet ownership on physical health is also well documented. The presence of pets can render peoples' perception of the surrounding environment as more friendly and less threatening, which ultimately may contribute to several positive physical health outcomes such as lowered blood pressure and heart rates (4–6). Dog walking is also negatively correlated with the body mass index, activities of daily living limitations, doctor visits and positively correlated with the vigorous exercise of the elderly (7). Moreover, pet ownership is a protective factor against allergy by increasing house dust bacterial diversity, reducing fungal species in the living environment, and strengthening the immune system (8, 9).

Since pets sometimes get sick like humans, the veterinary healthcare system is critical for pets' welfare. Previous studies have documented various barriers to seeking veterinary services, such as cost, operation hours, geographic location, transportation, educational attainment of pet owners, culture, language, and veterinarian-owner communication (10–13). The difference in native languages between veterinarians and owners may prohibit effective communication for trust-building between owners and veterinarians, which can affect a pet owner's willingness to consult a veterinarian (12). Among all barriers, care cost has been identified as the dominant factor determining owners' decision on seeking veterinary services (10, 11, 13–15). Financial constraints of pet owners may negatively impact their decisions of seeking a veterinarian and potentially impair their pets' welfare (10). A previous study in the United States (U.S.) indicates that the 2007–2009 economic recession was the primary factor that drove the growing concern of the cost of veterinary services because of potential job or income loss (14). The study also found that unemployed or low-income pet owners were less likely to consult a veterinarian than full-time employed or higher-income counterparts (14). The decision to seek a veterinarian is made with reference to various factors, including but not limited to, clinical symptoms (e.g., trauma, ingested poisonous substances, and end-of-life care), a pet owner's income level, cost barriers, and transportation barriers for seeking veterinary services as well (11). An animal welfare study conducted in Soweto, Gauteng, South Africa, found that <1% of the respondents used private vehicles while more than 60 and 30.5% relied on taxis and mobile services provided by the Society for the Prevention of Cruelty to Animals (SPCA) to seek veterinary services in the low-income urban community (16). Though this study did not further investigate how cost and transportation interact to affect animal welfare, this study's finding provides an important implication that cost plus the availability of affordable transport options may affect how the pet is delivered.

Spatial accessibility, concerning the interaction between supply, demand, and mobility, defines the ease of travel and the spatial variation of availability (17–20). The concept of spatial accessibility to everyday services or facilities, such as supermarkets, public bikes, health, and medical services, has been extensively studied for human health, and social and spatial inequality of such services, in many cases, has been widely identified (21–25). For instance, a Geographic Information System (GIS) study on the accessibility of supermarkets in London, Ontario, Canada, found that neighborhoods in an inner-city with low socioeconomic status have the poorest access to supermarkets. Such social and spatial inequality has been exacerbated over time, raising the concern of access to healthy and affordable food for the underprivileged (24). While it is not uncommon to use GIS in veterinary studies, few studies, to our understanding, apply it to assess the spatial or spatial-temporal accessibility of everyday veterinary services (26). Several studies on the accessibility of veterinary clinics identified transportation as one of the major hurdles to accessible veterinary services (11, 13, 27), but they mainly employed questionnaires from a user's subjective perspective without objective quantitative analysis on spatial accessibility of veterinary services. Spatial accessibility is critical because veterinary services beyond acceptable walking distance potentially limit available veterinary services. In addition, companion animals are often prohibited in public transport in many countries, causing transportation costs to escalate when people resort to private transport or taxis as they are the only travel option (12, 27). Therefore, a study on spatial variation of veterinary services is essential to identify underserved groups, which may provide a reference for future planning of veterinary services.

Since major public transport systems, such as Mass Transit Railway (MTR) and buses, prohibit animals in Hong Kong, the most feasible travel modes are private cars or taxis. When the distance to veterinary service is beyond walking distance, Hong Kongers without cars would have to rely on taxis and private cars, thus incurring an increased transportation cost. Furthermore, the uncertainty of the traffic condition such as gridlock and “change shifts” of the taxi drivers may also lead to a delay in proper veterinary treatment. From 2005 to 2016, the number of registered veterinary surgeons and pet dogs and cats increased from around 400 and 297,000 to around 800 and 511,000, respectively (28). In 2010, the availability of veterinarians defined by the veterinarian-to-pet ratio in Hong Kong was 1:735, far higher than Singapore (1:2,543), the U.S. (1:3,072), and the United Kingdom (1:2,374) (28). However, an increase in the overall supply of veterinary services does not guarantee an increase in the spatial accessibility of the services for every area and pet owner. The underserved area will likely remain unserved if additional veterinary clinics cluster only at a specific geographic locale. The literature on pet welfare or veterinary services is sparse in Hong Kong and is mainly confined to users' perceptions and practices, such as satisfaction with the veterinary services, the intention of relinquishment, and vaccination, without considering the travel impedance or spatial accessibility (28, 29). Therefore, there is a need to investigate the spatial accessibility of veterinary services in Hong Kong.

This paper aims to examine the spatial accessibility of veterinary services and their implications to social and spatial inequality in Hong Kong by using GIS. This paper will be one of the first studies that incorporate spatial data and geospatial technology into companion animals' welfare and social and spatial inequality, providing a framework of how companion animals' welfare can be safeguarded and investigated through interdisciplinary approaches.

## Materials and Methods

The present study applies GIS and R programming to analyze the spatial accessibility of veterinary services at the district level in Hong Kong. This study sets the following questions: (i) How are general and 24/7 veterinary clinics in Hong Kong spatially distributed across its territory? (ii) What are the supply and demand ratios for general and 24/7 veterinary clinics for each district in Hong Kong? (iii) What may contribute to the spatial availability of general and 24/7 veterinary clinics? (iv) Can general and 24/7 veterinary clinics be reachable within walking distance in Hong Kong? (v) Is there a social and spatial inequality of accessibility of veterinary services?

### Study Area

Hong Kong, a Special Administrative Region of China, is a metropolitan city in the eastern Pearl River Delta in Southeast China. As of mid-2020, a population of 7.48 million resides in Hong Kong, formulating 2.6 million households with a median monthly household income of 28,200 Hong Kong dollars (HKD) as of the first quarter of 2020 (3,617USD equivalent) (30). Hong Kong occupies a 1,114 km^2^ land area, of which 25.1% is densely urbanized while 70.3 and 4.6% are natural landscape and agricultural land, respectively (31). The population density of Hong Kong is 6,715 people per square kilometer for its whole territory and 26,751 people per square kilometer for its urbanized land area (279.6 km^2^). There are 18 District Council Districts (hereinafter districts) in Hong Kong, and we performed our analysis at the district level in this study. The district is an important small area unit for administration and council election and is a most commonly used boundary for public communication in Hong Kong.

**Figure 1:**
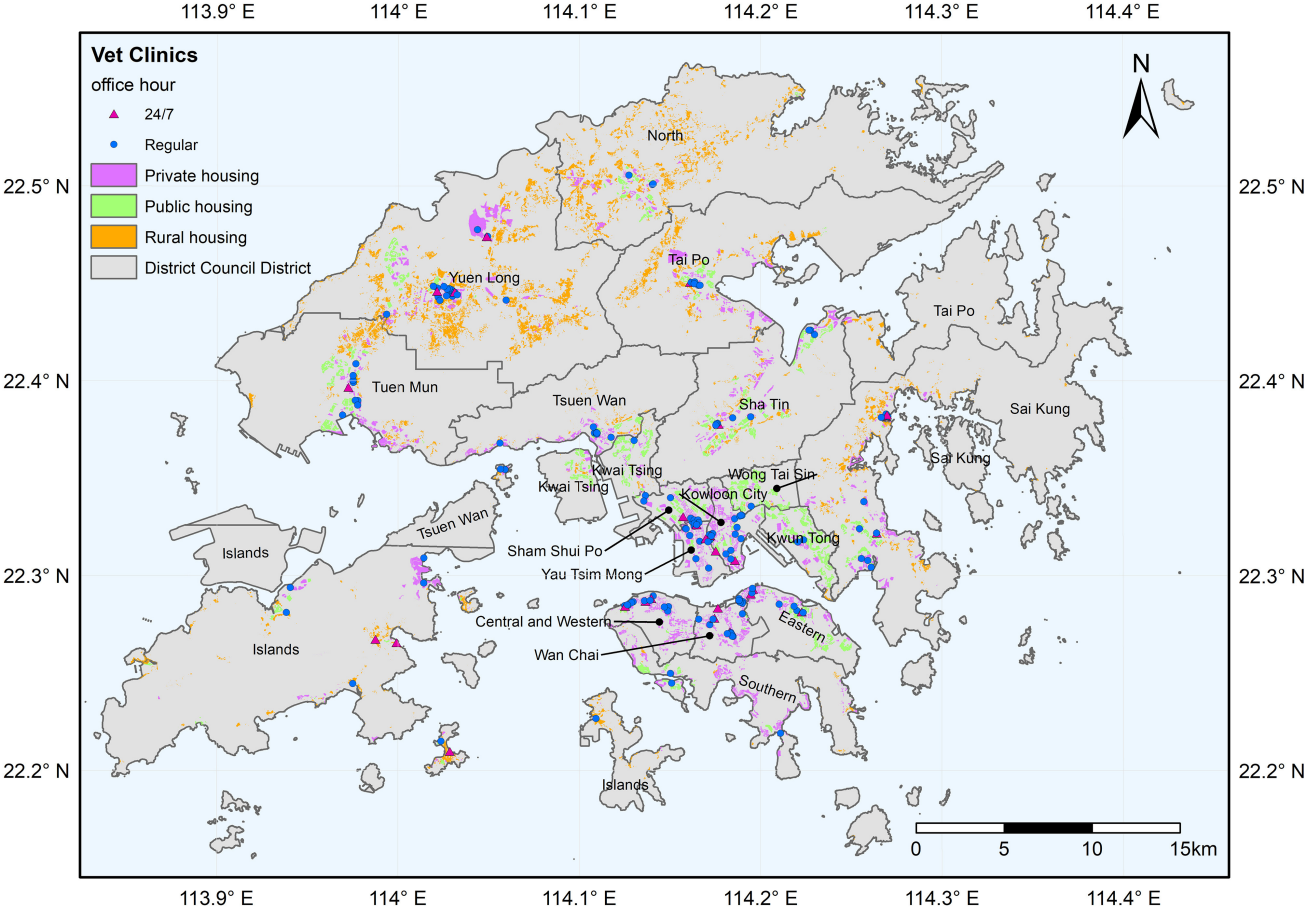
Spatial distribution of general and 24/7 vet clinics and the residential types in Hong Kong.

The Kowloon Peninsula and the north of Hong Kong Island are the traditional urban core where urbanization first took place. In response to population growth, the population was redistributed away from the core to the new growth area, such as Sha Tin, northern Island, Tuen Mun, and Sai Kung Districts (32). The new growth area is comprised mostly of government-led new towns and many private developments (32). Therefore, as shown in Figure 1, housing in the traditional urban core, such as Wan Chai, Yau Tsim Mong, and Eastern Districts are mostly private housing, while the new growth area is composed of much public housing and private housing. Northernmost Hong Kong, such as Yuen Long and North Districts and some outlying islands, such as Island District, are less dense and less developed, featuring extensive rural housing land use built with village houses (Figure 1). Public housing (as defined in the land use map in Figure 1) can be divided into public rental housing and subsidized home ownership housing. Public rental housing provides accommodation with affordable rental for low-income families who cannot afford the rental of private housing (33). Subsidized home ownership Scheme sells flats for low- to middle-income families at prices cheaper than the property market (33). As of the first quarter of 2020, 30.8, 14.6, and 53.9% of all domestic households in Hong Kong live in public rental housing, subsidized homeownership housing, and private permanent housing, and earn a median household income of 18,000, 28,000 and 40,000HKD, respectively (30).

### Data

Data used in this study encompass the addresses and office hours of the veterinary clinics, land utilization map, census data, the Hong Kong coastline, and district map. We first obtained the latest list of veterinary clinics (last updated on July 23, 2020) from the Hong Kong Agriculture, Fisheries, and Conservation Department website. Duplicated records and clinics with no address were removed. Then, we obtained the office hours of each clinic from each clinic's website. Roof-top geocoding was performed by using Google Earth Pro. There are 185 veterinary clinics, of which 30 provide 24/7 services (Figure 1).

The 2020 land utilization map, on 10 x 10 m grids, produced by compiling satellite images, governmental surveys, and records, was obtained from the website of the Hong Kong Planning Department (31).

The latest census data (2016 version) were obtained from the Hong Kong government's GeoData Store website. This dataset is in a GIS shapefile comprising 18 districts and bounded by administrative boundary. The Hong Kong coastline was obtained by clipping the global land area by the Hong Kong administrative boundary. The global land area on the Open Streetmap (OSM) was downloaded from https://osmdata.openstreetmap.de/info/license.html. The Hong Kong administrative boundary was obtained from the plugin “Quick OSM” in QGIS. The final product of the census data is a polygon shapefile bounded by the coastline (Figure 1). The number of households in the census data was used to proxy the demand for veterinary services.

### Data Processing and Analysis

**Figure 2:**
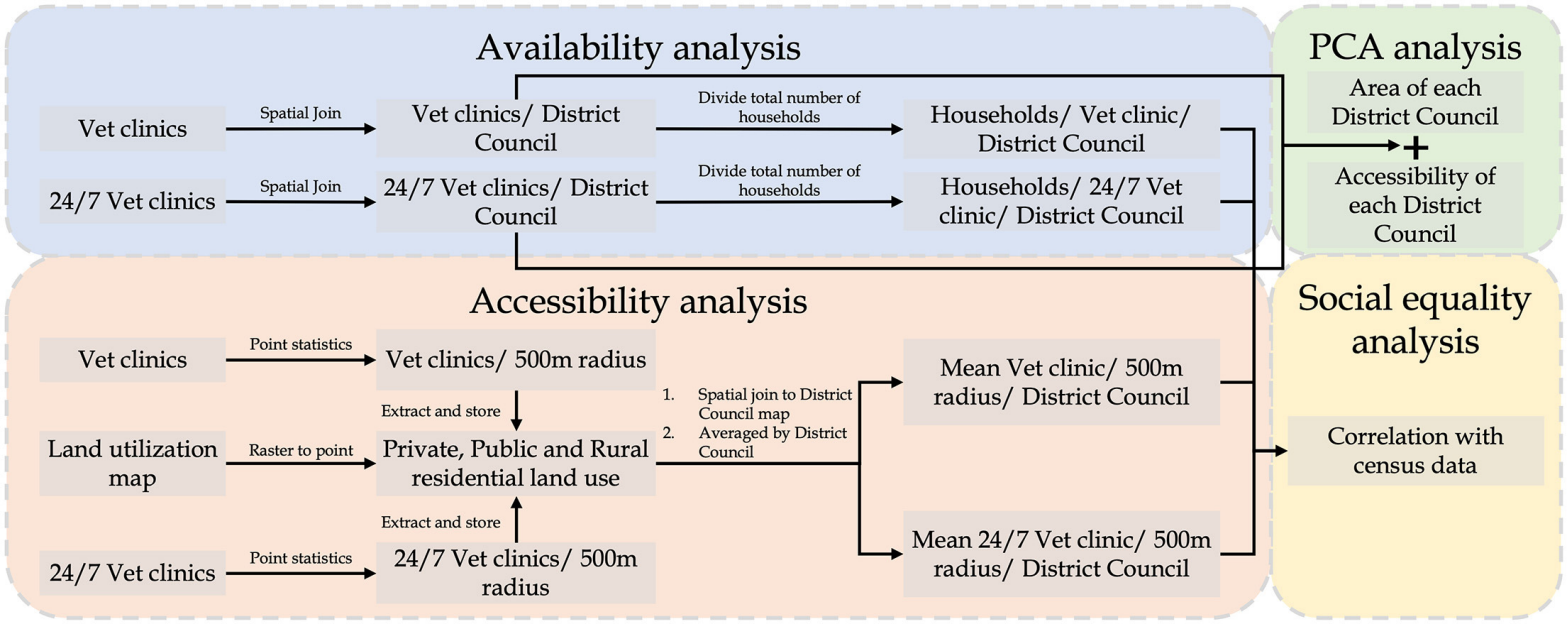
Flow chart of the data processing and analysis of this study.

Figure 2 presents four components of this study's analysis and the data processing workflow. Spatial accessibility *per se* considers the number of options available in a given space, while service availability is also affected by the demand that may lead to competition and the shortage when demand outweighs supply. Therefore, the availability analysis will first investigate the supply of and potential demand for veterinary services per district to identify the potential service gap. Hereinafter, *general vet clinics* refer to all veterinary clinics, and the *24/7 vet clinics* are those veterinary clinics providing 24-h services every day or providing emergency services after office hours. *Second*, a Principal Component Analysis (PCA) was conducted to investigate the potential factors leading to the distribution pattern of vet clinics. PCA is a powerful dimensionality-reduction technique widely used for unsupervised machine learning to reduce the data's dimensionality while minimizing information loss and providing valuable data classification information (34). *Third*, the accessibility analysis investigates the number of general and 24/7 vet clinics available within walking distance (500 m). Since the level of urbanization varies by district, averaging the general and 24/7 vet clinics available within walking distance for each district could be biased by its share of land use and the area. Therefore, this study confined the computation of accessibility into the residential area to avoid bias to land use and area. Only private, public, and rural residential land uses were retained in the land utilization map (Figure 1). Finally, the relationship between the pattern of socio-demographic characteristics and spatial availability/accessibility was explored. We used R for statistical analysis and ArcGIS for the geospatial analysis of this study.

## Results

### Potential Service Gap and Accessibility of Veterinary Services

**Figure 3:**
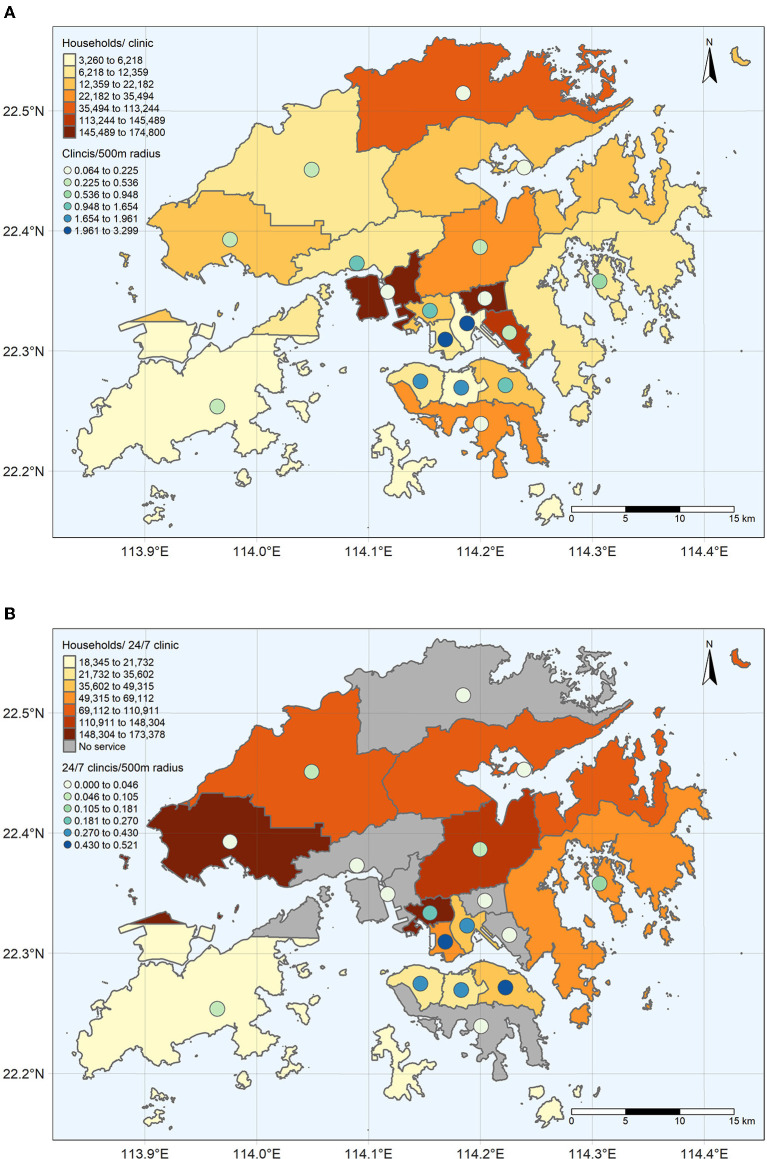
The average number of **(A)** general and the **(B)** 24/7 vet clinics per 500 m radius in residential area per district (point) superimposed on the number of households per **(A)** general and **(B)** 24/7 vet clinic for each district in Hong Kong (polygon). The Natural Breaks classification method is used to determine the interval breaks.

Supply and demand for veterinary services determine the availability of services spatially and temporally. Figure 3 shows the number of households per vet clinic for each district. Kwai Tsing, Wong Tai Sin, and Kwun Tong Districts experience the most supply-demand deficit, where each vet clinic may serve over 113,244 households (Figure 3A). In contrast, districts such as Wan Chai, Islands, and Kowloon City serve the least number of households (6,218 or less) (Figure 3A).

Besides, the supply-demand gap widens when considering services out of regular working hours, suggesting spatial and temporal inequalities (Figure 3B). There are no 24/7 veterinary services available in North, Tsuen Wan, Kwai Tsing, Wong Tai Sin, Kwun Tong, and Southern Districts. The number of households served by 24/7 vet clinics in the Islands, Central and Western, and the Wan Chai Districts is the least (35,602 households or less), while Tuen Mun and Kwai Tsing serve the most (148,304 households or more). A greater number of households served by the 24/7 vet clinics than general vet clinics is expected as fewer clinics provide 24/7 services, which may be due to higher operating costs and fewer emergency cases than regular cases. However, 24/7 veterinary services are not available in every district, which makes cross-district travel unavoidable for people living in districts without a 24/7 vet clinic to consult the veterinarian under an emergency condition beyond office hours.

Regarding the spatial accessibility of the services, the numbers of general vet clinics within walking distance are the highest in the northern Hong Kong Island, Kowloon City, and the Yau Tsim Mong Districts (Figure 3A). On average, people living in these districts may gain access to one or more general vet clinics within walking distance. In contrast, Southern, Kwai Tsing, Wong Tai Sin, North, and Tai Po Districts have fewer general vet clinics within 500 m walking distance (0.064–0.225 vet clinics per 500 m radius), implying that most residents in these communities may require a vehicle ride or long walking journey to its nearest clinic.

The pattern of access to 24/7 vet clinics within 500 m walking distance (Figure 3B) generally resembles the pattern of general vet clinics (Figure 3A) that the highest average number of 24/7 clinics within a 500 m radius is found in northern Hong Kong Island, Kowloon City and the Yau Tsim Mong Districts. However, the number of available 24/7 vet clinics within walking distance is much fewer than the general vet clinic. For instance, the highest number for general vet clinics is 3.299, while it drops to 0.521 for 24/7 vet clinics. It suggests that, generally, vet clinics may not be reachable within walking distance in most of the residential areas after regular office hours.

### Relationship Between Area, Average District-to-District Distances, and the Distribution of Veterinary Services

**Figure 4:**
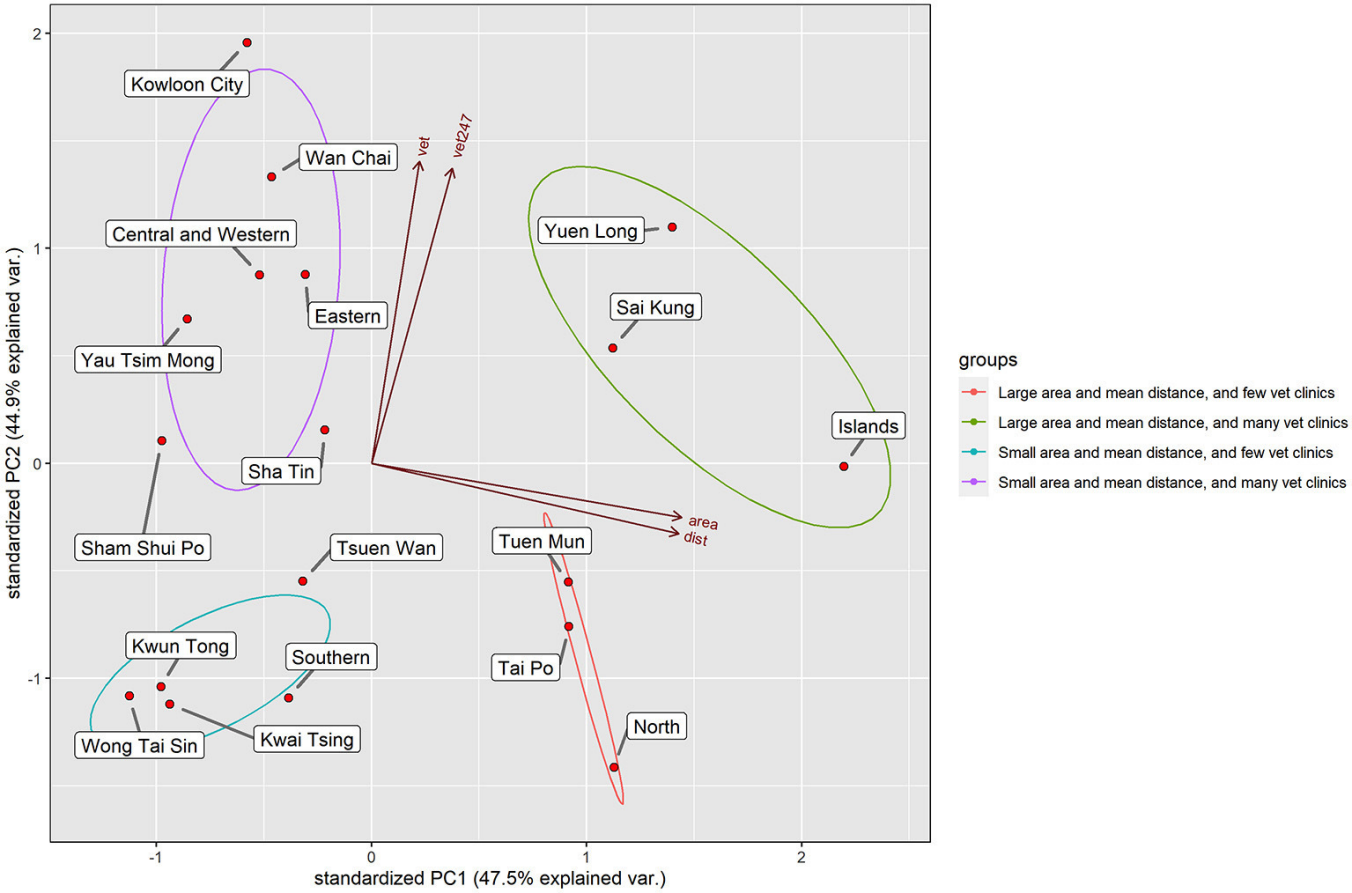
The biplot of the reprojected values on PC1 and PC2. The arrows are the eigenvalues and eigenvectors of each variable.

While we have identified a clustering pattern of vet clinics and unequal access to veterinary services, it is unsure what underlying factors may be related to this spatial distribution of vet clinics. According to the *central place theory* in geography, the location choice of a store rests on two essential concepts—i.e., the inner and the outer ranges (35, 36). The outer range defines the maximum distance the customers are willing to patronize, while the inner range defines the radius of the area from the store that contains the necessary demand to support the store. A store can only be economically viable when the outer range is greater than the inner range because the more-than-necessary demand can be secured in the catchment area (35). Therefore, its attractiveness highly depends on the ease of travel and the store's catchment area. We created a distance matrix of the average district-to-district distances from a district to all the other districts to proxy the accessibility of each district, which is calculated by averaging the Euclidian distance from the centroid point of each district to the centroid points of all the other districts. However, the average district-to-district distances are not significantly correlated with the number of general vet clinics (P>0.05) and 24/7 vet clinics (P>0.05) for each district. This indicates that the distribution of the vet clinics may not be only related to the overall accessibility of the districts linearly. Furthermore, the average district-to-district distances do not take the regional variation and size of the district into account. Therefore, this paper, as illustrated in Figure 4, employed the PCA to explore the relationship between average district-to-district distances (presented as *dist*), area (presented as *area*), and the number of general vet clinics (presented as *vet*) and 24/7 vet clinics (presented as *vet247*).

**Figure 5:**
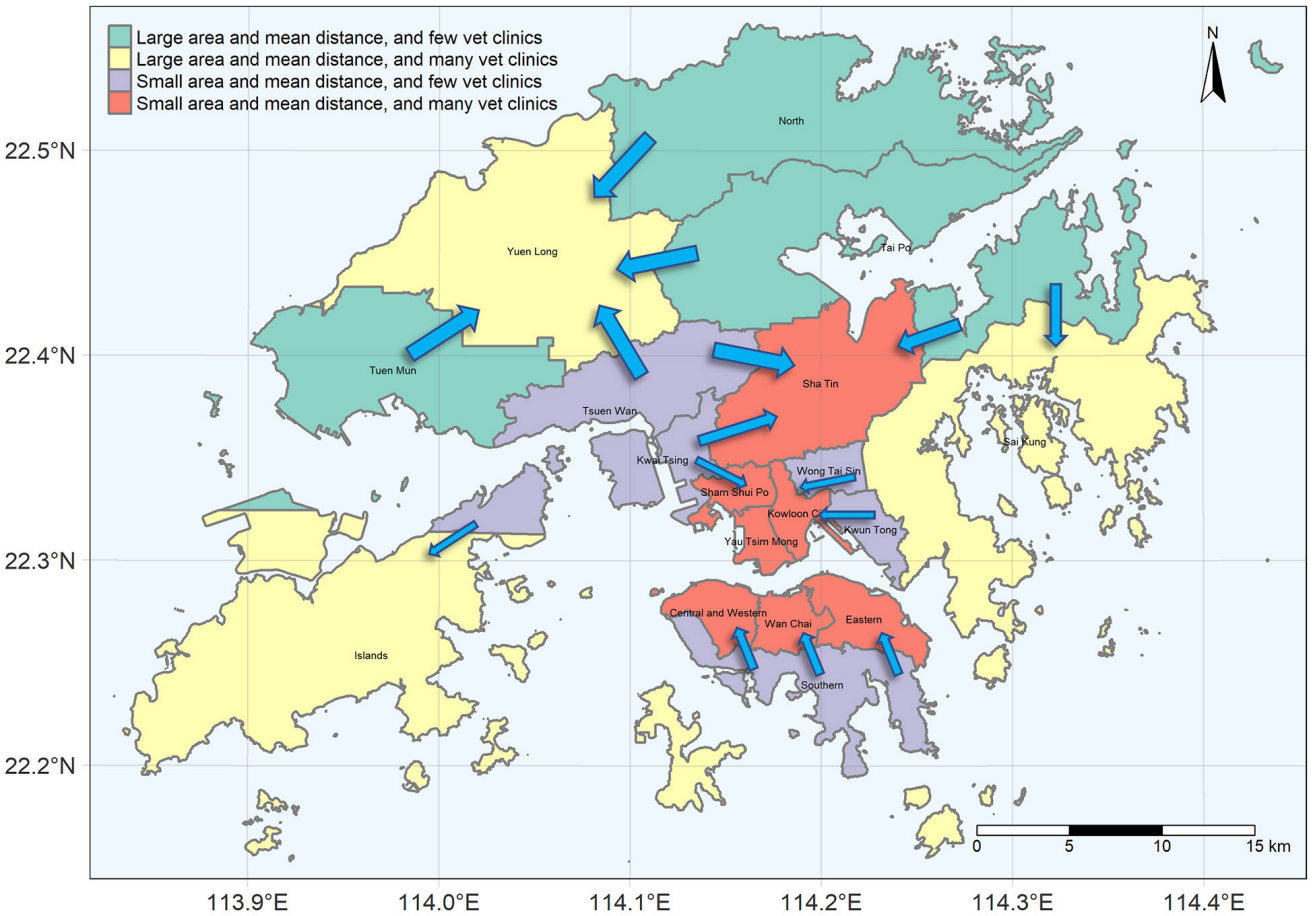
Classification map derived from the PCA in Figure 4. Arrows indicate the movement of customers.

The four input variables produced four principal components (PCs), known as the data dimensions. The first two PCs explained 92.46% of the total variance, so the first two PCs contained the most useful information. Four clusters can be identified from the biplot of the PC1 and PC2 (Figure 4), and they were used to produce a nominal map (Figure 5). As shown in Figure 5, Yuen Long, Sha Tin, Sham Shui Po, Kowloon City, Yau Tsim Mong, Island Districts, and the districts in the northern Hong Kong Island are higher in the availability of vet clinics. However, Tuen Mun, North, Southern, Wong Tai Sin, Kwun Tong, Tsuen Wan, Tai Po, Kwai Tsing Districts are lower in the availability of vet clinics. From the pattern shown in Figure 5, we can observe that districts with few vet clinics (districts in green and in purple) are immediately adjacent to the district with many vet clinics (districts in red and in yellow). Therefore, the arrows (Figure 5) show the direction of customer flow if vet service is not sufficient (or a 24/7 vet clinic is not available). Overall, one cross-district movement is required for districts with few vet clinics to reach the districts with many vet clinics. The lower average district-to-district distances for a district suggests that travel cost is lower than other districts and indicates that the district is more accessible among the entire territory. Caution should be taken that the average district-to-district distances capture the spatial centrality of the districts, and it provides information on the level of spatial accessibility at the district level. Owing to the shorter average district-to-district distances, vet clinics tend to concentrate in Yau Tsim Mong, Sham Shui Po, Kowloon City and Sha Tin Districts, and the Northern Hong Kong Island. The ease of travel both within and across districts may provide convenience to staff and customers, increasing its attractiveness to recruit staff and attract customers. Since Island and Sai Kung Districts have a large area and are located at the outermost of the Hong Kong territories, their travel time within/between the districts is relatively longer. Therefore, these districts may have an isolated market for many vet clinics, more targeting local patients within the districts. The northernmost part of Hong Kong has a relatively low average district-to-district distance but with a larger area such as Yuen Long, Tuen Mun, and North Districts. As Yuen Long District is large in area and is located between Tuen Mun and North Districts, more vet clinics may serve here for a relatively large within-district market. As a result, the distribution of vet clinics may be related to the accessibility, area, and market.

### Social and Spatial Inequality of Accessibility to Veterinary Services

The distribution of vet clinics and 24/7 vet clinics and the space-time supply-demand gap have been investigated above. This section investigates the implication of the spatial-temporal availability of veterinary services to social equality.

**Figure 6:**
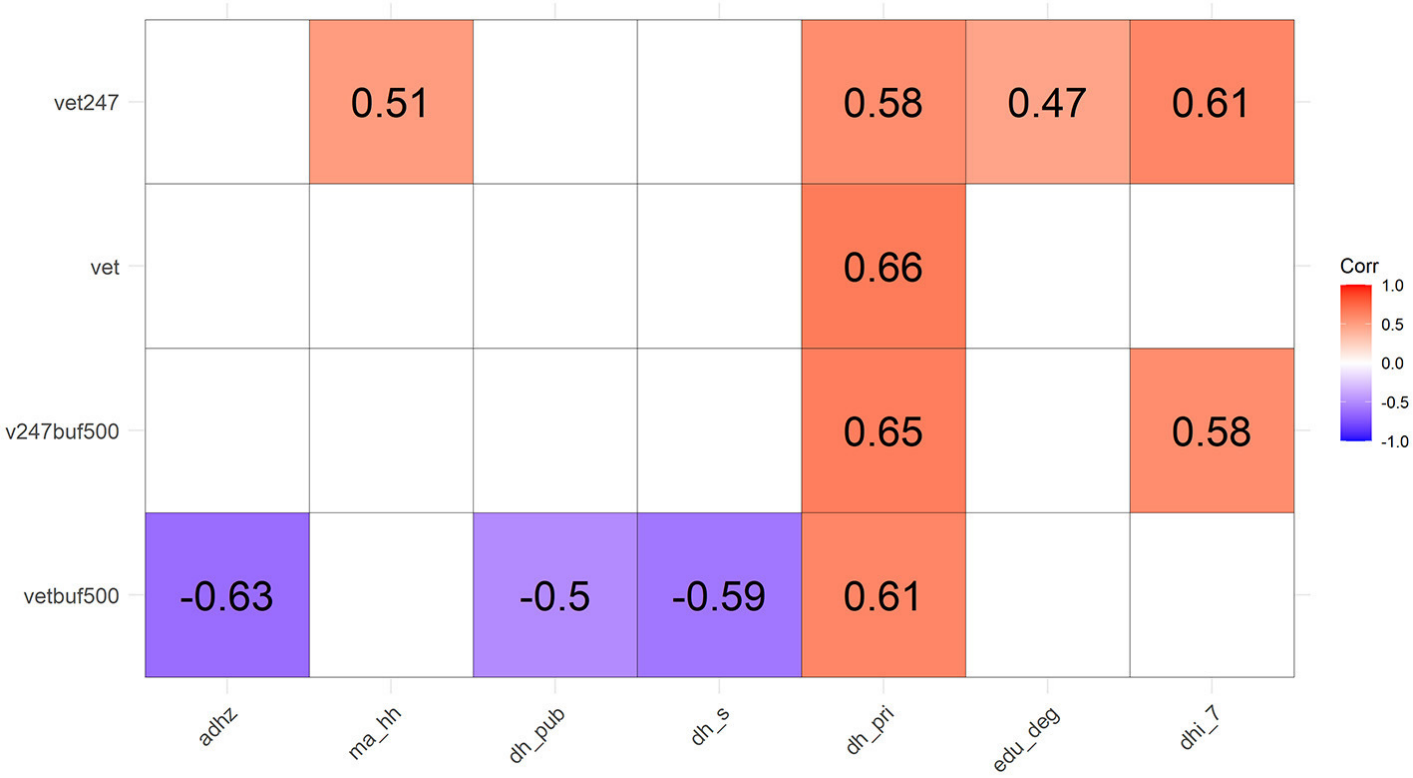
Correlation matrix of accessibility and availability of general and 24/7 vet clinics with demographic characteristics. Correlation with a *P*-value >0.05 is left blank.

Figure 6 shows the correlations of spatial accessibility and availability of general and 24/7 vet clinics with demographic characteristics. When considering the availability of general (*vet*) and 24/7 (*vet247*) vet clinics per district, they significantly correlate with the number of domestic households living in private housing (*dh_pri*). This suggests that districts with more private-housing households have more general and 24/7 vet clinics. Furthermore, the availability of 24/7 vet clinics also positively correlates with the median monthly domestic household income (*ma*_*hh*), population with a post-secondary degree (*edu_deg*), and households with monthly domestic households income ≥60,000HKD (*dhi_7*; the highest income category in the census record). This implies that vet clinics offering 24/7 services may tend to locate in the district with a larger population with higher educational attainment, the wealthiest households, more private-housing households, and higher median monthly domestic household income.

The spatial accessibility of general (*vetbuf500*) and 24/7 (*v247buf500*) vet clinics positively correlates with the number of households living in private housing, which is statistically significant at 0.05 level. It is consistent that availability at the district level and within walking distance positively correlates with private housing households (statistically significant at 0.05 level). The accessibility of general vet clinics also negatively correlates with the average domestic household size (*adhz*), and the number of public housing households (*dh_pub*) and subsidized homeownership households (*dh_s*) (all are statistically significant at 0.05 level). Overall, districts with higher availability of general vet clinics per 500 m radius in residential areas may have a smaller average domestic household size and less public and subsidized housing households while having more private housing households. Conversely, the accessibility of 24/7 vet clinics also significantly correlates with the number of households with monthly households income ≥60,000HKD. Therefore, it may suggest that a community with more wealthiest households may be more accessible to the 24/7 vet clinics within walking distance. Other than ≥60,000HKD income category, none of the other income categories (≤ 6,000; 6,000–9,999; 10,000–19,999; 20,000–29,999; 30,000–39,999; 40,000–59,999) significantly correlated with accessibility and availability of general or 24/7 vet clinics.

**Figure 7:**
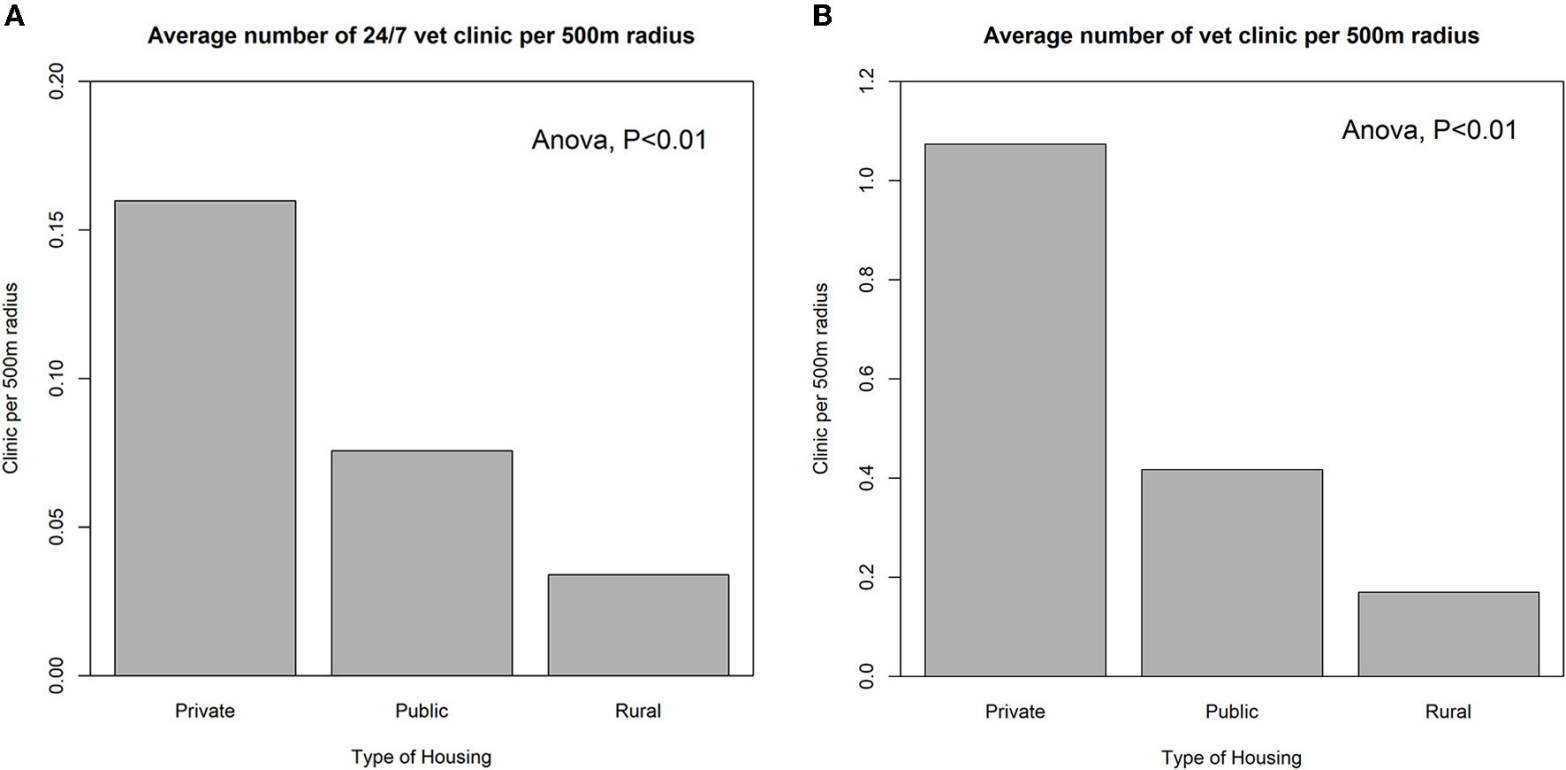
The average number of **(A)** general and **(B)** 24/7 vet clinics by housing type per 500 m radius per 10 m × 10 m grid in the residential areas.

Spatial accessibility of veterinary services was also examined with residential types. We averaged the number of general and 24/7 vet clinics per 500 m radius per grid in the residential area by housing type (Figure 7). On average, people living in private housing can reach 1.07 vet clinics, which is the greatest among all housing types (Public: 0.42; Rural: 0.17) (Figure 7A). This difference is statistically significant at 0.01 level according to the ANOVA test. As expected, the difference in the number of 24/7 vet clinics accessible among residential types is identical to the general vet clinic, while the reachable number of clinics drops substantially (Private: 0.160; Public: 0.08; Rural: 0.03; *P* < 0.01 according to ANOVA test) (Figure 7B). Age and marital status were also incorporated into the correlation analysis, but no significant relationship was found.

## Discussion

This study provides an exploratory analysis of how the provision of veterinary services varies across space and time and its implication to social equality by using advanced geospatial data techniques.

Employing PCA, we found that the area and the average district-to-district distances may be related to the locational decision of vet clinics. Stores tend to locate in areas where a sufficient population can visit within acceptable travel distance and be economically viable (37). In addition, as the minimum differentiation theory and the principle of cumulative attraction suggest, stores with similar features may be economically viable when they are arranged in a clustered geographic order than in a dispersed manner (37). A cluster of stores with a similar nature can provide wider choices within a restricted area and hence, it may reduce the uncertainty and search cost for the customers, increasing the market's overall attractiveness (36, 37). This may address why the vet clinics cluster spatially (Figure 1).

Apart from the physical environment as revealed in the PCA analysis, the demographic characteristics may also alter the spatial-temporal provision of veterinary services, further revealing the underlying business consideration and social inequality. This study discovered that general and 24/7 vet clinics are the most available and accessible to private housing than public and rural housing. Besides, household income, as well as educational attainment, is also positively related to the accessibility and availability of 24/7 vet clinics in a district. A previous study has indicated that private-housing households dominate the number of pet ownership in Hong Kong: 85 and 63.8% of households that owned cats and dogs are living in private housing, respectively; and 14.9 and 36.2% of households that owned cats and dogs are living in a public rental or subsidized homeownership housing, respectively (29). This suggests that private housing households may constitute the major market share of veterinary services. People with higher income are more likely to have a pet (29) and have fewer financial constraints to veterinary care (38). Moreover, the positive influence of educational attainment on visiting vet clinics rests on a better awareness of their pets' medical needs (13) and more promising employability (38). Besides, accessibility to general vet clinics is negatively associated with household size (Figure 6). A previous study found that over 40% of households keeping dogs/cats in Hong Kong have only one to two household members (29). Further study is required to investigate the relationship between pet ownership and household size, while the negative association between the household size and accessibility to general vet clinic may relate to the covariation with pet ownership. Moving to the perspective of clinic operation, veterinarians with the heavy education debt load, productivity-based remuneration system of a clinic, together with logistical, operational, and financial challenges, may discourage the setup of vet clinics in remote or rural areas (38). Therefore, vet clinics may target a more affluent and educated population to cover the most active group of customers. However, this market-oriented practice may consequently facilitate unequal access to veterinary services (13).

In Hong Kong, the public transport system is mature, facilitating low car dependency (39, 40) and achieving around 90% of the passenger trips using public transport (41). Railways and franchised buses are the most popular transit mode (75.1%) (Table 1), but they explicitly prohibit animals (42). Riding with animals on Public Light Buses are at the drivers' discretion, but they are low in capacity (16 passengers per vehicle). Furthermore, Public Light Buses serve as the supplementary transport mode providing services for the area where the market is too small to support franchised buses service or for the area with low accessibility (44). Therefore, Public Light Buses cannot provide universal service, and its low capacity may also make it uncertain whether the owner can reach vet clinics on time. Pets are also allowed by some ferry operators. For instance, each pet is charged 9.5 to 20.5HKD for routes operated by Sun Ferry Services Company Ltd (43). However, some of the additional costs are comparable to the base charge of taxis (from 19 to 24HKD). Ferry is a less popular travel mode in Hong Kong (Table 1), where the railway is centered as the backbone of the public transport system (44). Under the circumstance that the transit system is very convenient and cheap, people in Hong Kong mainly rely on public transit for their daily travel. Taking a taxi is the most viable option for seeking veterinary services, especially in an urgent situation, as it provides point-to-point and high flexibility services and minimizes the required walking journey. Therefore, the transport option is limited, and the distance to veterinary services may lead to additional expenditure other than veterinary consultation costs.

**Table 1:** Public Transport Patronage in 2019 and animal-friendly policy by Mode.

**Transport mode**	**Thousand patronages (%)**	**Allow animal (except guide dog)**	**Additional charge**
Railways	1,917,359 (42.2)	No	/
Franchised buses	1,494,283 (32.9)	No	/
Public light buses	642,796 (14.2)	On driver'/ operators' decision	No
Taxis	311,945 (6.9)	On driver's decision	Yes
Residents' services	77,989 (1.7)	No data	/
Ferries	44,593 (1)	Varies among operators	Vary
MTR buses	51,484 (1.1)	No	/

*Source: (40, 42, 43)*.

Those living in the public rental or subsidized homeownership housing feature lower income and low private car ownership (32). Therefore, they can be sensitive to transportation costs and consultation costs. Though this research found that households living in rural housing (combined with the private housing household by the government in the census data) have the lowest accessibility, they have higher income and also higher car ownership (32). Therefore, those living in public housing could be more sensitive to transportation and cost. These suggest that those who are wealthier (and more sophisticated) have greater accessibility to general (and 24/7) veterinary services than those who are less financially viable. Therefore, less affluent people may take more time and cost for accessing veterinary services. Future studies should conduct a micro-scale study on the barriers and difficulties of taking a taxi to seek a veterinarian to justify this hypothesis further. The cost barriers plus financial constraints may reduce the pet owners' willingness to seek veterinary services, increasing the risk of their pets developing serious diseases due to delay in seeking treatment (14, 27). Furthermore, the financial burden on raising pets may also increase the risk of pet relinquishment (45, 46). A previous survey conducted in Hong Kong found that 52.6% of respondents who had considered relinquishment attribute the reason to financial problems (29). Hence, the cost of raising pets, such as transportation, veterinary cost, and daily necessity, is one of the factors to be emphasized in animal welfare issues.

To relieve the financial burden, both from consultation costs and transportation, low-cost and/or mobile services can be provided for the underserved community. Many low-cost or free veterinary services have been established in many countries such as Canada (47), the U.S. (27, 48), and the United Kingdom (49). In Hong Kong, SPCA and Non-Profit making Veterinary Services Society (NPV) have provided reduced-cost, mobile services for the outlying areas (50, 51). These services may alleviate the financial burden for the underserved population and promote the equity of medical support. For instance, the SPCA mobile clinic serves around 3,000 companion animals annually (50). However, the SPCA suspended the mobile clinic since 2020 owing to operational reasons, while the NPV mobile clinic only provides service 2 days a week (50, 51). This may incur a threat to animal welfare as the service may become more competitive and those who cannot afford the regular price may delay seeking medical services until their pet deteriorates to a serious condition.

The implication for future veterinary services from this paper's findings is twofold. First, focusing areas in the spotlight may overlook areas otherwise in need. For instance, the mobile clinics, operated by both SPCA and NPV, provide services in remote areas, such as Tuen Mun, Island, and the North Districts (50, 51). However, some new growth areas, such as Wong Tai Sin, Southern, and Kwai Tsing Districts, are close to the traditional urban core but are also poor in accessibility to veterinary services (Figures 3A,B). Therefore, the need-based veterinary services should also consider areas not geographically remote but are indeed the service desert. Second, need-based services should also extend beyond regular office hours. To our understanding, low-cost veterinary service is rare and only NPV provides both low-cost and 24/7 services (51, 52). However, the NPV is situated in Eastern and Yau Tsim Mong Districts, while only the latter provides 24/7 service (51, 52). The limited spatial-temporal coverage of low-cost services, plus the focus on the remote area, may create a service gap in new growth areas close to the traditional urban core.

Due to this paper's findings, the authors call for a comprehensive situational analysis of Hong Kong's animal welfare through qualitative and quantitative approaches. This paper found that geographically accessible new growth areas may be overlooked in the need-based service system and we cannot accurately estimate the number of the needy. To sustain and optimize the need-based services, future study should estimate the number of the needy and provide a clearer picture of the service gap and lay down a direction for service planning. For instance, a public health study on adult obesity in the U.S. used survey data to simulate the prevalence of obesity at the county level by using spatial microsimulation techniques (53). Future studies may consider this technique to estimate the actual demand for veterinary services. In addition, we also need to investigate how other factors, such as transportation, consultation cost, and language barriers, may affect owners' decision on seeking veterinarians, which is important to identify how to improve the overall service system to safeguard the companion animal's welfare.

This study provides an empirical framework for carrying out cross-disciplinary analysis on animal welfare and an essential account of the strategic planning of veterinary services. Yet, there are some limitations of this research. This paper used household numbers rather than the actual pet counts to investigate the supply-demand gap due to data unavailability. This method may yield potential errors as it does not account for some contextual factors related to pet ownership, such as the prohibition of raising pets in the property and limited room for raising pets. Furthermore, this study does not account for the clinic's capacity, such as the number of veterinarians on duty, reputation, services provided, and open hours. Therefore, it may pose some potential errors in estimating the supply-demand gap. However, this may not affect our conclusion on the spatial accessibility of veterinary services because we focus on the distance and travel impedance over space and time rather than whether the demand can be totally met.

## Conclusion

Using GIS, this study examined the spatial-temporal accessibility of veterinary services and their implication to social and spatial inequality in Hong Kong. Our study implies that there may exist spatial-temporal inequalities in accessibility of veterinary services, potentially worsening animal welfare in Hong Kong. We also argue that the need-based veterinary support tends to target remote rural areas while overlooking the new growth areas close to the traditional urban core but poor in the accessibility of veterinary care. Therefore, a comprehensive investigation into the pet ownership landscape and their needs over space and time will be beneficial to construct a more robust protection net for animal welfare in Hong Kong.

## Data Availability Statement

Data used in this paper are publicly available and the source is disclosed in this paper. The processed data supporting the analysis of this article can be made available by the authors on request.

## Author Contributions

KN: conceptualization, methodology, software, formal analysis, investigation, data curation, writing—original draft, writing—review and editing, and visualization. CH: conceptualization, writing—original draft, and writing—review and editing. KK: conceptualization, methodology, writing—original draft, writing—review and editing, supervision, project administration, and funding acquisition. All authors contributed to the article and approved the submitted version.

## Funding

This study was partially funded by the University of Hong Kong Faculty of Social Sciences' Preemptive Retention Fund and the Hui Oi-Chow Trust Fund awarded to KK.

## Conflict of Interest

The authors declare that the research was conducted in the absence of any commercial or financial relationships that could be construed as a potential conflict of interest.

## Publisher's Note

All claims expressed in this article are solely those of the authors and do not necessarily represent those of their affiliated organizations, or those of the publisher, the editors and the reviewers. Any product that may be evaluated in this article, or claim that may be made by its manufacturer, is not guaranteed or endorsed by the publisher.
